# An integrative review: maternal engagement in the neonatal intensive care unit and health outcomes for U.S.-born preterm infants and their parents

**DOI:** 10.3934/publichealth.2019.2.160

**Published:** 2019-05-05

**Authors:** Susanne Klawetter, Jennifer C. Greenfield, Stephanie Rachel Speer, Kyria Brown, Sunah S. Hwang

**Affiliations:** 1 School of Social Work, Portland State University, Portland, OR, USA; 2 Graduate School of Social Work, University of Denver, Denver, CO USA; 3 Graduate School of Social Work, University of Denver, Denver, CO, USA; 4 Graduate School of Social Work, University of Denver, Denver, CO USA; School of Public Health, University of Colorado, Aurora, CO, USA; 5 School of Medicine, University of Colorado; Department of Pediatrics, Children's Hospital Colorado, Aurora, CO, USA

**Keywords:** maternal engagement, neonatal intensive care unit, NICU, preterm birth, integrative review

## Abstract

Hospitals and perinatal organizations recognize the importance of family engagement in the neonatal intensive care unit (NICU). The Agency for Healthcare Research and Quality (AHRQ) defines family engagement as “A set of behaviors by patients, family members, and health professionals and a set of organizational policies and procedures that foster both the inclusion of patients and family members as active members of the health care team and collaborative partnerships with providers and provider organizations.” In-unit barriers and facilitators to enhance family engagement are well studied; however, less is known specifically about maternal engagement's influence in the NICU on the health of infants and mothers, particularly within U.S. social and healthcare contexts. In this integrative review, we examine the relationship between maternal engagement in the NICU and preterm infant and maternal health outcomes within the U.S. Results from the 33 articles that met inclusion criteria indicate that maternal engagement in the NICU is associated with infant outcomes, maternal health-behavior outcomes, maternal mental health outcomes, maternal-child bonding outcomes, and breastfeeding outcomes. Skin-to-skin holding is the most studied maternal engagement activity in the U.S. preterm NICU population. Further research is needed to understand what types of engagement are most salient, how they should be measured, and which immediate outcomes are the best predictors of long-term health and well-being.

## 1. Introduction

Approximately 10% of infants born in the United States (U.S.) are premature, defined as gestational age < 37 weeks [1]. Unlike their healthy term counterparts, preterm infants remain hospitalized for a prolonged period of time [2]–[4]. The quantity and quality of time that mothers can spend in the care of their preterm infants during this critical period is affected by individual and institutional factors [5],[6]. At the individual level, mothers have varying degrees of knowledge, resources or supports, expectations, and confidence levels around actively engaging in the care of their infants. At the broader health systems level, medical providers, hospital practices, and policies may influence mothers' ability to participate in infant care.

While a clear definition of family engagement in the neonatal intensive care unit (NICU) is lacking, the Agency for Healthcare Research and Quality (AHRQ) conceptualized family engagement in the following way in their Guide to Patient and Family Engagement: “A set of behaviors by patients, family members, and health professionals and a set of organizational policies and procedures that foster both the inclusion of patients and family members as active members of the health care team and collaborative partnerships with providers and provider organizations.” [5]. Family engagement in the NICU is recognized as a priority by hospitals and academic and public health perinatal organizations, and the in-unit barriers and facilitators to optimizing family engagement have been well studied internationally [7]–[10]. However, less is known specifically about maternal engagement's influence in the NICU on the health of infants and parents, particularly within U.S. social and healthcare contexts. Fathers and other family members undoubtedly have important engagement in the NICU. However, maternal engagement is unique in its relationship to breastfeeding, maternal-infant bonding/attachment, and the pregnancy, delivery, and postpartum experience. Consequently, this integrative review focuses specifically on maternal engagement in U.S. NICUs.

While not undertaken in the U.S., investigators from Canada, Europe, Australia and New Zealand recently published results from their multicenter cluster-randomized controlled trial that assessed the effect of family integrated care in the NICU on infant and parent outcomes, safety, and resource use [11]. The intervention included parental presence at the infant bedside for at least 6 hours per day, attendance at educational sessions, and active participation in infant care. In this study of 26 sites, compared to infants who received standard care, infants in the family integrated care (FICare) group had better weight gain during birth hospitalization and exclusive breastmilk feeding at discharge. Parents in the FICare group had lower mean stress scores and lower mean anxiety scores. There were no significant differences in the following infant health outcomes: mortality, major morbidity, duration of oxygen therapy, and duration of hospitalization.

Although the studies included in this integrative review demonstrate interest in investigating the relationships between maternal engagement in NICU care and infant and maternal health outcomes, research such as this FICare study remains difficult to implement in the U.S. This country is one of the few around the world with no universal parental leave program; while 60% of U.S. mothers are eligible for up to 12 weeks of job-guaranteed, unpaid leave through the Family and Medical Leave Act, a large number of eligible mothers do not take this leave because of financial constraints. Furthermore, many parents who are offered paid leave through their employers receive far less than the leave available to parents in other countries. A few states in the U.S. now mandate access to paid leave for new parents, but wage replacement rates in these programs are often lower than the full income needed for parents to take advantage of a lengthy leave. For instance, California's paid leave program offers, on average, 55% of the worker's weekly income for up to six weeks; New York's paid leave program, effective January 1, 2018, will be more generous when it is fully implemented in 2022, but will offer up to 12 weeks of leave at a wage replacement rate of only 67% [12]. Under these conditions, it is difficult to implement a robust randomized-control trial of interventions that require significant time commitments by parents of hospitalized preterm infants such as the FICare study. Nonetheless, findings from studies conducted outside the U.S. may be informative as the policy debate around paid leave continues to intensify in the U.S..

Despite these obstacles, it is important to understand the presence and magnitude of the potential benefits to infants and mothers from greater family engagement. In the full-term population, numerous studies have demonstrated that optimizing maternal-infant bonding leads to improved behavioral and developmental outcomes. In fact, when maternal interaction with her infant is disrupted through depression, anxiety, or stress, infants are at greater risk for cognitive and behavioral problems [13],[14]. Given that preterm infants are at greater risk for adverse medical and developmental outcomes, it is crucial to understand whether enhancing maternal engagement in the NICU may present a strategy for reducing both short-term and longer-term health risks.

This integrative review synthesizes the literature related to maternal engagement with preterm infants in U.S. NICUs. Because we wanted a comprehensive snapshot of current empirical knowledge about the impact of maternal engagement on infant and maternal physical and mental health, we searched as far back into the literature as peer-reviewed studies of our key constructs allowed. We build on the AHRQ definition of engagement to include behaviors that reflect interactions between parents and their hospitalized infant(s) such as NICU visitation, skin-to-skin or kangaroo care holding, traditional holding, infant massage, music stimulation, and interventions designed to facilitate maternal-infant interaction and bonding through psychoeducation and various combinations of the aforementioned activities. While the focus of this review is on maternal engagement, some studies include both mothers and fathers in the study sample. Studies only including fathers are excluded, but studies that include either mothers exclusively or both mothers and fathers are included in this review. Different etiologies lead to NICU hospitalization, which in turn leads to unique sets of challenges to maternal engagement in the NICU. Due to the prevalence of preterm birth resulting in NICU hospitalization, this manuscript reviews literature related only to maternal engagement and premature infants in the NICU. Finally, all studies included in this review are conducted in U.S. NICUs. Numerous studies examine maternal engagement in the NICU around the world, however, the U.S. presents a unique healthcare and social context that shape care practices and maternal engagement and cause the U.S. NICU context to be distinctly different from NICUs in other countries. We believe a current review of the literature regarding maternal engagement with preterm infants in the U.S. will aid in identifying research gaps and ultimately in delivering best NICU practice.

## 2. Methods

### 2.1. Databases and search methodology

To understand how maternal engagement with preterm infants in U.S. NICUs has been examined in the literature, a search was conducted using the Preferred Reporting Items for Systematic Reviews and Meta-Analyses (PRISMA) as a guide [15]. The PubMed and PsycINFO electronic databases were used to identify peer-reviewed articles that met inclusion criteria for this review. Search terms were: *kangaroo care, kangaroo mother care, skin-to-skin, maternal visiting, maternal visitation, parental visiting, parental visitation, maternal holding, maternal engagement*, NICU, and *neonatal intensive care unit*. No specific dates of publication parameters were set; however, the final database review was conducted on November 1, 2018, so no articles published in the database after that date are included in this review. Only articles written in English are included.

### 2.2. Article selection

Articles were included if they were original research with data collected in the U.S., included preterm infants (broadly defined as those born at 37 gestational weeks or less) and their parents or mothers, examined parental engagement-related activities in the NICU as a dependent variable, and explored maternal, parental, or infant health outcomes as independent variables. Studies that examined only paternal engagement or paternal health outcomes were excluded, given this review's focus on maternal engagement. Using the search terms, 358 abstracts were identified through the PubMed electronic database and an additional 3 abstracts from reference list reviews. After screening the total 361 abstracts, 270 were excluded for not meeting inclusion criteria.

**Figure 1.**
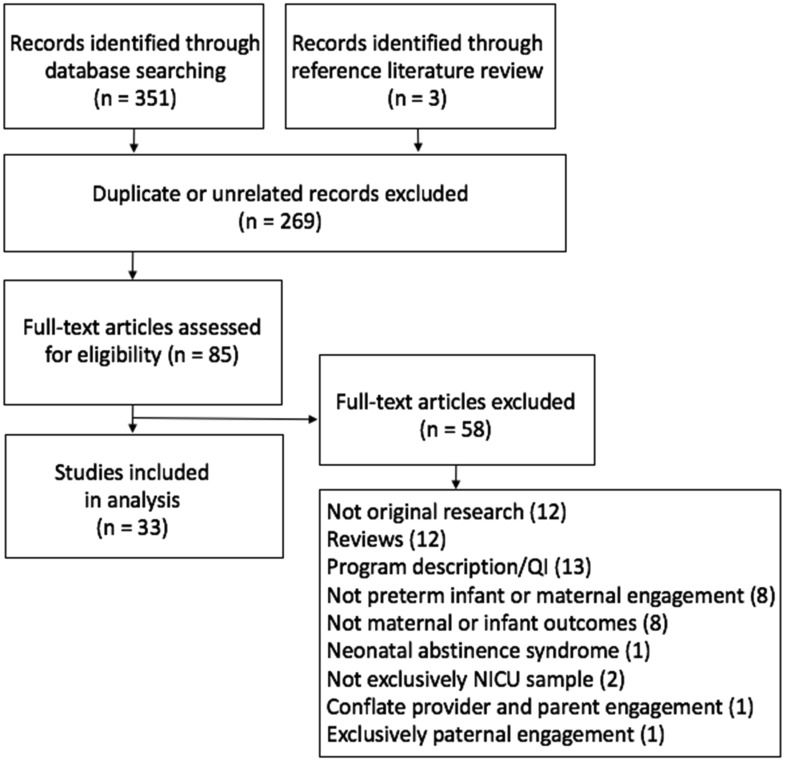
Article selection process.

The remaining 91 articles were screened in-depth and 58 were subsequently excluded for not being original research (12), being reviews of studies (12), being descriptions of programs or quality improvement projects (13), not examining preterm infants or maternal engagement activity (8), not including maternal or child outcomes as a variable (8), examining neonatal abstinence syndrome in the infant sample (1), study not conducted exclusively in the NICU (2), conflating provider and parent engagement (1), and exclusively paternal engagement (1). There were 33 remaining articles that met criteria for inclusion in this review (Figure 1).

All 33 articles were reviewed thoroughly by two members of the research team. Articles focused on maternal engagement-related activities or behaviors including NICU visitation, skin-to-skin or kangaroo care holding, traditional holding, infant massage, music stimulation, and interventions designed to facilitate maternal-infant interaction and bonding through psychoeducation and various combinations of the aforementioned activities. While all articles included in this review included the examination of maternal engagement-related activities, the literature currently does not provide a clear definition for behaviors and activities that reflect what the authors would call “maternal engagement.” Ideally, the literature would reflect a consistent definition or operationalization of this construct, especially given the growing emphasis on family-centered care practices in the NICU.

Articles also examined maternal or infant health/mental health outcomes as dependent variables. Multiple articles examined more than one health/mental health outcome domain. For example, a study could explore the relationships among skin-to-skin holding, infant health outcomes, and maternal mental health outcomes. We have chosen to organize our presentation of findings through the categorization of outcomes studied in the articles we review; although it would also be interesting to group the articles by type(s) of engagement studied, we found that the lack of specificity and consistency in definitions of engagement would make this more difficult. Therefore, we first offer some general commentary and examples of how studies have conceptualized maternal engagement, then present more specific and in-depth analysis of the studies in a discussion that is grouped according to themes that emerged in our analysis of the outcomes measured by these studies. These categories include 1) infant health outcomes, 2) maternal health-behavior outcomes, 3) maternal mental health outcomes, 4) maternal-child bonding outcomes, and 5) breastfeeding outcomes. Although we provide some noteworthy details about sampling and methods for each studied cited, we also summarize the studies with greater detail in Table 1.

**Table 1.** Summary of articles included in the integrative review: maternal engagement in U.S. NICUs.

Author/Date	Title	Research Question/Aim	Design	Methods	Sample	Results/Conclusion
Affonso et al., 1993	Reconciliation and healing for mothers through skin-to-skin contact provided in an American tertiary level intensive care nursery	To determine the feasibility and safety of SSC through KC as well as any effect on the mother's emotional care.	Qualitative	Mothers participated in three sets of semi-structured interviews after KC.	N = 8 mother/preterm infant dyads, infant weight between 1,250 and 1,750 g, 24–32 weeks GA.	Qualitative themes of reconciliation and healing when mothers placed their infant STS over three consecutive weeks.
Bloch-Salisbury et al., 2014	Kangaroo care: Cardio-respiratory relationships between the infant and caregiver	To investigate physiological relationships between the infant/caregiver during SSC, and if respiratory stability of the premature infant is influenced by the caregiver's heartbeat.	Prospective cross-control study	Infant physiological markers were collected during SSC and a control period.	N = 11 parent/pre-term infants (GA < 35 weeks); mean PCA 32 weeks.	During SSC, infant respiratory instability and apnea incidence were each related to care giver heartrate variance instead of directly related to their own heartrate variance as in the control.
Cong et al, 2009	Kangaroo care modifies preterm infant heart rate variability in response to heel stick pain: Pilot study	To determine if KC results in improved balance in autonomic responses to heel stick pain than the standard method where infants remain in IC for the heel stick.	Prospective randomized cross-over trial (two group)	Infant physiological markers and reactions to routine heel sticks (using ABSS) were collected during 24 hours of KC-inclusive care and 24 hours of routine IC.	N = 14 mother/preterm infant dyads, infants 30–32 weeks GA and less than 9 days postnatal age.	Infants experienced better balance in response to KC than IC condition as shown by more autonomic stability during heel stick, KC may be helpful in mediating physiologic response in preterm infants.
Cong et al., 2012	Effects of skin-to-skin contact on autonomic pain responses in preterm infants	To determine the effects on autonomic responses in preterm infants for 30 minutes sessions of KC, 15 minutes of KC, and IC before and throughout heel stick.	Prospective randomized cross-over trial (three group)	Infant physiological markers and reactions to routine heel sticks (using ABSS) were collected during KC for durations of 30 minutes, 15 minutes, and IC.	N = 26 mother/preterm infant dyads, infants 28 to less than 32 weeks GA and less than 14 days postnatal age.	This study showed that KC has a significant effect on reducing autonomic pain responses in preterm infants. The findings support that KC is a safe and effective pain intervention in the NICU.
Cong et al., 2015	Parental oxytocin responses during skin-to-skin contact in pre-term infants	To determine if oxytocin modulates parent stress and anxiety during maternal and paternal SSC with preterm infants.	Prospective randomized cross-over trial (two group)	The effects of KC on the mother's and father's hormone levels were observed pre-KC, during KC, and post-KC. Parents self-rated their anxiety during each stage.	N = 28 Parent/preterm infant triads, infants were stable and between 30 to less than 35 weeks GA, and 3–10 days postnatal age.	SSC activated the oxytocin release and reduced stress and anxiety responses in mothers and fathers of pre-term infants. SSC plays a positive role in early post-partum period and patterns of maternal and paternal bio-behavioral responses to SSC with pre-term infants might be different.
Gale et al, 1993	Skin-to-skin (kangaroo) holding of the intubated premature infant	To determine if SSC affects visitation, attachment or caregiving behaviors among substance abusing mothers.	Mixed methods; Prospective cohort and qualitative	Demographics and physiological information was gathered. Parents were interviewed concerning their NICU and KC experiences.	N = 25 Parent/intubated infant dyad/triads, infants of any GA (Range = 28–33 weeks GA at first holding).	This study suggests that STS with small intubated infants may offer some parents an effective method to overcome some of the barriers to attachment imposed by the infant's hospitalization.
Goyna et al., 2017	Investigating skin-to-skin care patterns with extremely preterm infants in the NICU and their effect on early cognitive and communication performance: A retrospective cohort study	To investigate how patterns of SSC might impact infant early cognitive and communication performance.	Retrospective cohort study	Medical record extraction. Multistep process of logistic regressions, t-tests, χ2 tests and Fisher's exact tests followed with exploratory network analysis using novel visual analytic software.	N = 97 extremely preterm infants, GA < 27 weeks who completed 6-month and 12-month developmental assessments in the follow-up clinic.	This study suggests an association between early and frequent skin-to-skin care with extremely preterm infants and early cognitive and communication performance.
Greene et al, 2015	Maternal psychological distress and visitation to the neonatal intensive care unit	To examine any association between maternal visitation, psychological distress, and preterm infant post-NICU discharge outcomes.	Prospective longitudinal and questionnaires	Distress questionnaires given at 1 month post birth, 2 weeks prior to discharge, post discharge at 4 month CA. Visitation in NICU was recorded, as well as infant clinic attendance and rehospitalization.	N = 69 mother/VBLW preterm infant dyads.	Distress is an important predictor of visitation. In turn, visitation is associated with long-term maternal distress.
Hane et al., 2015	Family Nurture Intervention improves the quality of maternal caregiving in the neonatal intensive care unit: Evidence from a randomized controlled trial	To determine the impact of FNI on maternal caregiving behavior.	Intervention study; Randomized control trial	Maternal caregiving behavior was coded during 30 minute holding and feeding periods prior to NICU discharge as an evaluation of FNI.	N = 65 mother/preterm infant dyads.	This is the first study to demonstrate that in-unit MCB can be enhanced by a hospital-based intervention. FNI provides a new rationale for integrating nurture-based interventions into standard NICU care.
Holditch-Davis et al., 2014	Maternally administered interventions for preterm infants in the NICU: Effects on maternal psychological distress and mother-infant relationship	To determine if ATVV and KC affect maternal distress and mother-infant relationship.	Intervention study; Randomized control trial (three group)	Mothers completed questionnaires at 2, 6, and 12 months CA. At 2 and 6 months, mothers were videotaped interacting with their infant.	N = 240 mother/preterm infant dyads, infants weighed < 1750 g at birth.	These findings suggest that as short-term interventions, KC and ATVV, have important effects on mothers and their preterm infants, especially in the first half of the first year.
Hurst et al., 1997	Skin-to-skin holding in the neonatal intensive care unit influences maternal milk volume	To evaluate the effect of early initiation of STS holding on lactation.	Prospective cohort (for study participants) and Retrospective cohort (for control)	A repeated-measures analysis of variance adjusting for baseline volumes (1 week after delivery) was used to evaluate the difference in milk volumes between STS and control groups.	N = 23 mother/preterm infant dyads.	STS holding of LBW infants in the early intensive care phase can result in a significant increase in maternal milk volume, thereby overcoming the frequently seen insufficient lactation experienced by these mothers.
Johnson, 2007	The maternal experience of kangaroo holding.	To describe the maternal experience of kangaroo holding premature infants in the NICU.	Qualitative, naturalistic inquiry design	Semi-structured, open-ended interviews conducted with mothers.	N = 18 mother/preterm infant dyads.	Results identify maternal experiences of and responses to kangaroo holding in the intensive care environment, leading to the increased understanding of the multifaceted advantages of kangaroo holding on maternal attachment behaviors.
Lau et al., 2007	Ethnic/racial diversity, maternal stress, lactation and very low birthweight infants	To compare maternal characteristics and psychological stress profile mothers with LBW infants and determine association between psychosocial factors, milk expression frequency, SSC, lactation performance.	Repeated questionnaire	Self-reported psychological questionnaires were given every 2 weeks after delivery over 10 weeks. Milk expression frequency, STS, and socioeconomic variables were collected.	N = 124 mother/preterm infant dyads, infants born between 26–29 weeks GA.	The response bias to self-reported questionnaires may not provide an accurate profile of maternal psychosocial profile. Lactation performance can be best enhanced with a multi-faceted intervention program including incorporating parental involvement in infant care and encouragement for frequent milk expression and STS.
Lester et al., 2016	18-Month follow-up of infants cared for in a single-family room Neonatal Intensive Care Unit	To determine whether the SFR-NICU is associated with improved 18-month neurodevelopmental outcome, especially in infants of mothers with high maternal involvement.	Prospective longitudinal study	Infants divided into high versus low maternal involvement groups over a period of 18-month follow-up. Covariates of maternal, infant, and medical characteristics were abstracted from medical records.	N = 216 mother/infant dyads, infants born < 30 weeks GA.	High maternal involvement is associated with improved 18-month neurodevelopmental outcome, especially in infants cared for in a SFR-NICU.
Lewis et al., 1991	Visitation to a Neonatal Intensive Care Unit	To examine and describe visitation patterns to a NICU as a function of neonatal and family variables.	Longitudinal study	Daily number of visits to NICU-admitted infants were logged. Health markers, physical conditions, and demographic information were extracted from medical records of the infants.	N = 164 mother/infant dyads, infant birth weight < 2001 g.	The greater the number of days with no visitors, the poorer the likelihood that the infant was brought to a 3-month follow-up clinic appointment.
Ludington-Hoe et al., 2004	Randomized control trial of kangaroo care: Cardiorespiratory and thermal effects on health infants	To determine the safety and effects on healthy preterm infants of three continuous hours of KC compared to standard NICU care by measuring cardiorespiratory and thermal responses.	Randomized control trial, pretest-test-posttest design	Cardiorespiratory and thermal responses during three inter-feeding intervals were observed consecutively on one day.	N = 24 healthy preterm infants nearing discharge, infants 33–35 weeks gestation at birth.	Mean cardiorespiratory and temperature outcomes remained within clinically acceptable ranges during KC. Apnea, bradycardia, and periodic breathing were absent during KC. Regular breathing increased for infants receiving KC compared to standard NICU care.
McCain et al., 2005	Heart rate variability responses of a preterm infant to kangaroo care	To examine the heart rate variability responses of one preterm infant to a KC experience with his mother.	Case study report	Case study report of a 35-week old preterm infant's behavior and heart rate variability when placed STS on his mother's chest.	N = 1 preterm infant	HRV was increased with fussy behavior in the open crib and decreased with sleep during KC. KC produced changes in behavior and HRV that are illustrative of decreasing stress.
Neu et al., 2000	The impact of two transfer techniques used during skin-to-skin care on the physiologic and behavioral responses of preterm infants	To compare the impact of two different transfer techniques used in SSC (nurse transfer and parent transfer) on physiologic stability and other descriptive measures of physiologic stability related to energy conservation in ventilated preterm infants during and after SSC.	Prospective cohort	Fifteen ventilated preterm infants were randomly assigned to receive either parent or nurse-to-parent transfer on the first of 2 consecutive days and the alternate method the following day.	N = 15 ventilated preterm infants, mean weight 1094 g.	Both transfer methods resulted in physiologic disorganization. However, during and after STS care, infants exhibited no signs of energy depletion.
Neu et al., 2009	Coregulation in salivary cortisol during maternal holding of premature infants	To examine coregulation between mothers and preterm infants in hypothalamic-pituitary-adrenocortical activity.	Exploratory study	Maternal and infant cortisol levels were obtained at beginning and end of holding. Coregulation was operationalized as less difference between maternal-infant cortisol levels immediately after holding as compared to before holding.	N = 20 mother/infant dyads, infants mean postconceptional age of 34.7 weeks and average postnatal age of 15 days.	A coregulatory relationship in cortisol levels existed between mothers and infants during holding, which was moderated by sound levels. Nurses in the NICU can facilitate the mother–infant relationship by promoting a quiet environment, particularly around mothers who are holding their infants.
Neu et al., 2013	Influence of holding practice on preterm infant development	To determine if nurse supported kangaroo holding of healthy preterm infants in the first eight weeks of the infant's life facilitates early behavioral organization and development.	Randomized control trial (three group)	KC and blanket-held groups received 8 weekly educational visits from a RN. Maternal holding diary kept. APIB was administered when infants were 40 to 44 weeks PCA.	N = 87 mother/preterm infant dyads, infants born between 32–35 weeks GA.	Infants in the kangaroo and blanket groups had more optimal scores than the control group in Robust Crying. When kangaroo holding is compared to blanket holding, both methods may provide equal early behavioral organization and developmental benefit to the infant.
Neu et al., 2014	Effect of holding on co-regulation in preterm infants: A randomized controlled trial	To determine whether kangaroo holding of healthy preterm infants over the first eight weeks of the infant's life would facilitate co-regulation in salivary cortisol between mother and infant.	Randomized control trial (three group)	Similar methodology as Neu et al., 2013.	N = 79 mother/preterm infant dyads, infants born between 32–35 weeks GA, with a mean postnatal age of 15 days.	Decreasing level of cortisol in mothers/infants suggest that holding promoted the expected decline in stress hormone levels. Supported holding methods did not differentially affect co-regulation compared to controls. Stress may need to be present in order for mothers/infants to demonstrate co-regulation in cortisol levels.
Phillips et al., 2012	Prevention of postpartum smoking relapse in mothers of infants in the neonatal intensive care unit	To reduce postpartum smoking relapse and prolong breastfeeding duration during the first 8 weeks postpartum in mothers who quit smoking just before/during pregnancy and have NICU-admitted infants.	Randomized control trial (two group)	In addition to weekly encouragement to remain smoke free and routine breastfeeding support, intervention group mothers were also given enhanced support for maternal-infant bonding and STS was encouraged.	N = 49 mothers of preterm infants in a NICU.	Interventions to support mother-infant bonding during a newborn's hospitalization in the NICU are associated with reduced rates of smoking relapse and prolonged duration of breastfeeding during the first 8 weeks postpartum.
Pineda et al., 2018	Parent participation in the neonatal intensive care unit: Predictors and relationships to neurobehavior an developmental outcomes	To define predictors of parent presence, holding/SSC in the NICU and investigate relationships between parent participation and early neurobehavior/developmental outcomes at age 4-5 years among preterm infants.	Prospective longitudinal cohort study	Parent presence and holding/SSC were tracked throughout NICU hospitalization. Neurobehavior at TEA/development at 4 to 5 years were determined using standardized assessments.	N = 81 parent/preterm infant triads, infants born on or before 32 weeks GA, enrolled within 1 week of life in NICU.	Social and medical factors appear to impact parent presence, holding, and STS care in the NICU. Parent holding is related to better developmental outcomes, which highlights the importance of engaging families in the NICU.
Reynolds et al., 2013	Parental presence and holding in the neonatal intensive care unit and associations with early neurobehavior	To investigate the effects of parents' visitation and holding frequencies on infant development in the neonatal period.	Prospective longitudinal cohort study	Daily visits/holding activity recorded from birth until TE, supplemented with medical record extraction. Initial assessment used a modified version of the NISS, and later assessment used the NICU NNAS.	N = 81 parent/preterm infant triads, infants born on or before 30 weeks GA.	Infants who were visited and held more in the NICU had differences in early neurobehavior by term equivalent, which supports the need for and importance of early parenting in the NICU.
Roller, 2003	Getting to know you: Mothers' experiences of kangaroo care	To reveal mothers' experiences of providing KC for their preterm newborns while still in the hospital.	Qualitative, transcendental phenomenology	Semi-structured interviews were conducted with mothers 1 to 4 weeks postpartum.	N = 10 mothers who provided KC for their preterm newborns, 32–36 weeks GA, weighing 1500–3000 g.	KC facilitates bonding and enhances maternal-infant acquaintance, even in the NICU environment. Mothers found that KC calmed them and their newborns.
Samra et. al., 2015	Effect of skin-to-skin holding on stress in mothers of late-preterm infants: A randomized controlled trial	To examine the effect of SSC on stress perception between mothers who provided SSC to their late-preterm born infants and mothers who provided blanket holding.	Longitudinal randomized control trial	Maternal stress and physiologic stability was measured using the Parental Stressor intervention and SCRIP assessment respectively. Demographic and other covariates were extracted from medical records and daily logs were kept.	N = 40 mother/late preterm infant dyads, infants born between 34 and less than 37 weeks GA.	Mothers who provide SSC may experience more stress related to a more facilitated progression in the mother and infant relationship. The relationship between increased stress and the number of hours of SSC holding warrants further investigation.
Tully et al., 2016	A test of kangaroo care on preterm infant breastfeeding	To test the effects of KC on breastfeeding outcomes in preterm infants.	Secondary analysis of a randomized control trial (three group)	The treatment groups were instructed in KC or ATVV intervention and the control group received only preterm infant care information.	N = 231 racially diverse mother/preterm infant dyads, infants born weighing < 1,750 g.	Mothers who practiced KC, regardless of randomly allocated group, were more likely to provide their milk than those who did not practice KC. As implemented in this study, assignment to the KC group did not appear to influence the measured breastfeeding outcomes.
Vittner et al., 2017	Increase in Oxytocin from skin-to-skin contact enhances development of parent-infant relationship	To examine changes that occur in infant and parent salivary oxytocin/salivary cortisol levels during SSC and whether SSC alleviates parental stress and anxiety while supporting mother–father–infant relationships.	Randomized crossover design	Parental anxiety levels and saliva samples were collected from infants/mothers/fathers for oxytocin/cortisol measurement before, during, and after SSC. Parent–infant interaction was examined prior to discharge via video.	N = 28 parent/preterm infant triads, infants born between 30 and less than 35 weeks GA, and 3–10 days postnatal age.	Facilitation of SSC may be an effective intervention to reduce parent and infant stress in the NICU. Findings advance the exploration of oxytocin as a potential moderator for improving responsiveness and synchrony in parent–infant interactions.
Vittner et al., 2018	Parent engagement correlates with parent and preterm infant oxytocin release during skin-to-skin contact	To examine relationships between parental engagement and salivary oxytocin and cortisol levels for parents participating in SSC intervention.	Randomized crossover design	Parental engagement was measured using PREEMI prior to hospital discharge. Saliva samples for oxytocin and cortisol levels were collected before, during, and after SSC.	N = 28 parent/preterm infant triads, infants born between 30 and less than 35 weeks GA, and 3–10 days postnatal age.	Significant relationships exist between parental engagement and salivary oxytocin and cortisol levels. Defining parent engagement facilitates identification of parent risks and needs for intervention to optimize preterm outcomes. The PREEMI can serve as a standardized instrument to examine parent engagement.
Welch et al., 2013	Randomized controlled trial of Family Nurture Intervention in the NICU: Assessments of length of stay, feasibility and safety	To assess the effect of FNI on length of stay in the NICU and the feasibility and safety of its implementation in a high acuity level-IV NICU.	Randomized control trial (two group)	FNI was implemented by nurture specialists trained to facilitate affective communication between mother and infant during specified calming interactions.	N = 150 mother/preterm infant dyads from 115 families, infants born between 26–35 weeks GA.	There was no significant effect demonstrated with the FNI intervention amount on the primary short-term outcome, length of stay. FNI can be safely and feasibly implemented within a level-IV NICU.
Whipple, 2000	The effect of parent training in music and multimodal stimulation on parent neonate interactions in the Neonatal Intensive Care Unit	To examine the effects of parent training in music/multimodal stimulation on the quantity/quality of parent-neonate interactions, weight gain, and length of hospitalization of premature/LBW infants in a NICU.	Experimental/control group design	Parents in the experimental group received approximately one hour of instruction in appropriate uses of music and multimodal stimulation. Parent-neonate interactions were observed in both groups.	N = 20 mother/preterm infant dyads, infants born before 37 weeks GA, weight < 2500 g.	Infant stress behaviors were significantly fewer and appropriateness of parent actions and responses were significantly greater. Parents with the intervention self-reported spending significantly more time visiting in the NICU. A post-discharge follow-up showed little difference between groups for parent-infant interactions in the home.
White-Traut et al., 2012	Frequency of premature infant engagement and disengagement behaviors during two maternally administered interventions	To compare the frequency of premature infant engagement and disengagement behaviors during two maternally administered interventions, the multi-sensory ATVV intervention and KC.	Randomized control trial (three group)	Mothers in the ATVV intervention or KC groups administered the intervention a minimum of three times weekly and were video recorded weekly during intervention sessions.	N = 26 mother/preterm infant dyads, infants born between 21–32 weeks GA, weight ranged from 1030–2693 g.	The ATVV intervention elicited more disengagement, trended toward more engagement and more potent engagement, subtle disengagement, and potent disengagement behaviors than did KC. The ATVV intervention may be an intervention to promote the infant's learning how to regulate engagement and disengagement behaviors.
Zeskind et al., 1984	Effects of maternal visitation to preterm infants in the Neonatal Intensive Care Unit	To investigate whether directing mothers to make weekly NICU visitation appointments would increase frequency of independent maternal visitation and affect maternal perceptions of the infant/infant's length of hospitalization.	Randomized control trial	Assessments of mothers' perceptions of their infants at the initiation of the intervention program, immediately following the first maternal visit, at discharge, and at a 6-week post-discharge follow-up.	N = 32 mother/preterm infant dyads, healthy infants born between 30–36 weeks.	Results suggest that the mother's greater contact and familiarity with her infant, as a result of increased visitation, resulted in more realistic observations of her preterm infant's behavior and may have facilitated the recovery of the infant.

Note: Abbreviations: ABSS—Anderson Behavioral Scoring System (assessment), APIB—Assessment of Preterm Infant Behavior (assessment), ATVV—Auditory Tactile Visual Vestibular (intervention), CA—Corrected Age, DMC—Dyadic Mutuality Code (assessment), FNI—Family Nurture Intervention (intervention), GA—Gestational Age, IC—Incubator Care, IMIS—Index of Mother-Infant Separation (assessment), KC—Kangaroo Care, LBW—Low Birth Weight, PCA—Postconceptional Age, PREEMI—Parental Risk Evaluation Engagement Model Instrument (assessment), NCAST—Nursing Child Assessment Satellite Training–Feeding Scale (assessment), NICU—Neonatal Intensive Care Unit, NICU NNAS—NICU Network Neurobehavioral Assessment Scale (assessment), NISS—Neonatal Infant Stressor Scale (assessment), RN—Registered Nurse, SCRIP- Stability of the Cardiorespiratory System in Preterm Infants score (assessment), SFR—Single Family Room, SSC—Skin-to-skin Care/contact, STS—Skin-to-skin, TE—Term Equivalent, TEA—Term Equivalent Age, VLBW—Very Low Birth Weight.

## 3. Results

### 3.1. Measurement of maternal engagement

This integrative review found multiple methodologies used to measure maternal engagement activities. Sixteen studies used randomized control or crossover trial designs and twelve others used other quantitative designs. One study employed mixed methods design, three used qualitative methods, and one used a case study design. Samples studied were primarily drawn from single hospitals, with seven studies using samples recruited across larger hospital systems or from several hospitals.

As mentioned above, there is little consensus in the literature reviewed on how maternal engagement should be conceptualized or measured. Some studies, in fact, do not set out to measure the concept, but rather seek to test the effects of a specific type of engagement as a health intervention. Our review showed that specific types of maternal engagement measured included activities such as skin-to-skin holding, visitation, traditional holding, infant massage, and music stimulation. These activities may be measured for their relationship to maternal and infant physical and mental health outcomes. For example, skin-to-skin holding is the most often studied parental engagement activity, with 24 out of the 33 articles in this review including it as a variable. Among the many ways it is studied, it is examined for possible association to lactation performance, infant heart rate variability, infant response to pain, maternal smoking cessation, infant hospital length of stay, and parental stress and anxiety.

Some studies attempt to evaluate the effects of various combinations of these activities on infant and maternal health. For example, mothers may be trained to provide music stimulation and/or infant massage, or they may receive instruction and psychoeducation related to maternal-infant bonding in conjunction with family support and encouragement to kangaroo hold, and provide eye contact and soothing communication with their infant. One study evaluated the association between the NICU built environment (i.e., single family room and open-bay design), level of maternal involvement., and 18-month developmental follow-up. In this study, maternal involvement was measured by number of days per week of kangaroo care, bottle or breast feeding, and maternal care.

### 3.2. Infant outcomes

Many of the reviewed articles reported findings related to the relationship between maternal engagement and infant health outcomes. For the present analysis, maternal-infant bonding and breastfeeding outcomes are included in their own categories. Overall, our review of the literature suggests that maternal engagement activities are safe for infants and may be associated with positive infant health outcomes. There are studies noted here, however, that found no association to infant health outcomes.

An early study of 15 preterm infants evaluated the safety of skin-to-skin care while transferring ventilated preterm infants from their isolettes to skin-to-skin holding [16]. Infants were randomly assigned to either nurse-to-parent transfer or parent transfer groups. Skin-to-skin holding was associated with stabilized oxygen saturation levels, stabilized heart rates, and maintained energy levels regardless of transfer methods [17]. In a randomized control trial of 27 preterm infants, those who were skin-to-skin held in the NICU had clinically acceptable cardiorespiratory and temperature ranges during skin-to-skin holding and had no apnea or bradycardia events [18]. Infants who were skin-to-skin held had increased regular breathing patterns compared to infants who received standard NICU care [18]. McCain et al. also found in a case study that a preterm infant in the NICU had more a more stable and consistent heart rate when they were skin-to-skin held compared to when lying in their crib [19]. A more recent study evaluated the safety of the Family Nurture Intervention, an intervention designed to enhance families' bonding and caregiving experiences in the NICU [20]. This intervention included skin-to-skin holding as one of its caregiving enhancement elements. Findings from a randomized control trial of the Family Nurture Intervention confirmed the intervention was safe and feasible, as evidenced by no increase in medical risk or change in level of acuity for preterm infants [20].

Skin-to-skin holding has also been evaluated as an analgesic. Two crossover trials showed that preterm infants who were skin-to-skin held during heel sticks had more stable heart rates and recovered faster as evidenced by lower heart rates compared to infants in incubators [21],[22]. When infants (n = 26) were held skin-to-skin, they spent more time in quiet sleep states compared to when they were in incubators [21]. These findings were consistent when the infants received only fifteen-minute periods of skin-to-skin holding [21].

Various maternal engagement activities in the NICU are also linked to positive neurobehavioral outcomes among preterm infants. Maternal visitation is associated with positive infant arousal and motor development outcomes. Specifically, high maternal visitation rates were associated with less arousal, higher quality motor movement, less hypertonia, more hypotonia, and less excitability in a longitudinal cohort study of 81 preterm infants in the NICU [23]. This same study demonstrated that traditional blanket holding (i.e., not skin-to-skin holding) was associated with less infant stress and less infant excitability [23].

Blanket holding and skin-to-skin holding are also linked to more optimal crying patterns and motor development. In a randomized control trial of 87 preterm infants, parents were assigned to either a group who received encouragement from nurses to hold or skin-to-skin hold or a group who did not receive such encouragement. Evaluations of crying patterns showed that infants whose parents received encouragement to hold or skin-to-skin hold were able to vigorously cry and then be calmed and consoled more quickly than those whose parents did not receive the encouragement [24]. In this same study, there was no significant difference in crying patterns between blanket held or skin-to-skin held infants. A study of 81 parent-infant triads tracked parental visitation, blanket holding, and skin-to-skin holding patterns and found that blanket holding and skin-to-skin holding was linked to better infant reflex development at term. In this same study, skin-to-skin holding was also associated with less asymmetry at term [25].

A randomized crossover study comparing preterm infant (n = 28) cortisol levels prior to skin-to-skin holding to those directly after skin-to-skin holding found that preterm infants' cortisol levels decreased from pre-to post-skin-to-skin holding [26]. Since cortisol may serve as a signal of stress, this study suggests that skin-to-skin holding decreases stress in preterm infants.

Studies produce mixed findings regarding the effect of maternal engagement on infant length of stay in the NICU. An early randomized control trial of 32 mother-infant dyads found that higher maternal visitation rates in the NICU was related to shorter infant length of stay [27]. A later study that evaluated the effects of a music and multimodal stimulation parent training found that preterm infants whose parents received the training had less stress and shorter length of stay compared a control group [28]. However, the Family Nurture Intervention described above did not affect infant length of stay in the NICU [20]. These results suggest further study evaluating maternal engagement and preterm infant length of stay in the NICU is needed.

Finally, multiple studies have evaluated how maternal engagement activities may impact post-discharge infant outcomes. For the two studies expressly studying the effect of maternal visitation on infant post-discharge outcomes, findings are mixed. Lewis et al. found in their longitudinal study of 164 maternal-infant dyads, which included medical record data extraction, interviews with mothers, and 3-month follow-up clinic data, that maternal visitation was positively related to attendance at 3-month follow-up infant clinic visits [29]. However, Greene et al. found more recently in a study of 69 mother-infant dyads that maternal visitation rates were not linked to infant outcomes at discharge or post-discharge follow-up [30]. Skin-to-skin holding appears to have somewhat complicated infant post-discharge results in the literature, suggesting a need for further study in this area. Skin-to-skin holding was associated with more desirable scores on infant social behaviors and developmental maturity tests up to 12 months corrected age in a randomized control trial conducted with preterm infants and mothers in 4 U.S. hospitals [31]. In a study that evaluated infant post-discharge outcomes among 123 preterm infants who had stayed in a single-family room (SFR) NICU compared to 93 infants who had stayed in an open-bay NICU, infants from both NICU environments with more “highly involved mothers” had higher receptive and expressive communication at 18-month follow up [32]. Maternal involvement was measured through electronic medical record extraction of documentation of maternal and infant characteristics including participation in skin-to-skin holding, breast and/or bottle feedings, and maternal care in the NICU. Infant post-discharge outcomes were particularly positive among infants from the SFR NICU environment whose mothers also showed high levels of maternal involvement [32]. While a retrospective cohort study of parental skin-to-skin holding of extremely preterm infants, defined by the study's authors as GA < 27 weeks, in the NICU showed a positive but nonsignificant relationship between parents who skin-to-skin held and infant communication development [33], the Family Nurture Intervention trial demonstrated improved cognitive and language outcomes at the 18 month follow-up assessment between intervention and control groups [34]. A longitudinal cohort study followed 81 parent-infant triads over 4–5 years after recording parental visitation, blanket holding, and skin-to-skin holding patterns while preterm infants were hospitalized in the NICU. This study found that skin-to-skin holding was associated with gross motor development, but that other parent engagement activities were not [25].

### 3.3. Maternal health-behavior outcomes

While most studies explored how maternal engagement-related activities relate to maternal mental health outcomes, infant outcomes, maternal-child bonding outcomes, and/or breastfeeding outcomes, one study exclusively examined maternal health-behavior outcomes. Phillips et al. explored the effect of an intervention designed to support and maintain maternal smoking cessation during and after NICU hospitalization [35]. Mothers (n = 49) were randomized into either a control group (n = 28) which included support for breastfeeding and smoking cessation encouragement or an intervention group. The intervention group (n = 21) added a maternal-infant bonding support element through skin-to-skin holding and information about recognizing and interpreting infant behavioral cues. Mothers in the intervention group were more likely maintain their smoke-free status 8 weeks postpartum compared to mothers in the control group, suggesting that certain maternal engagement activities may support positive maternal health-behavior outcomes.

### 3.4. Maternal mental health outcomes

Several studies addressed different facets of how engagement in the NICU relates to maternal mental health and well-being. An early mixed methods study of 25 sets of parents of intubated preterm infants found that parents had positive perceptions of skin-to-skin holding and believed skin-to-skin holding helped them identify with and experience a sense of knowing their infants [36]. A qualitative study of 8 mothers of preterm infants in the NICU found that mothers who participate in skin-to-skin holding reported feelings of confidence, a sense of control, and competence [37]. These findings are echoed in a qualitative study (n = 18) in which mothers who skin-to-skin held felt confidence, described as feeling “needed” and “being comfortable” despite the health conditions of their infants [38]. More recently, a qualitative study of 10 mothers with preterm infants in the NICU affirm the value of skin-to-skin holding in helping them experience more self-awareness and maternal identity [39].

The literature also reflects how maternal engagement in the NICU relates to symptoms of depression and feelings of worry, stress, and anxiety. In a study comparing NICU visitation rates among 69 mothers, those with lower visitation rates were more likely to have had prior trauma exposure, low anxiety, and to experience symptoms of depression at their infants' 4 months corrected age [30]. In addition to visitation, skin-to-skin holding and activities that promote other forms of maternal-infant physical contact are linked to positive maternal mental health and well-being. In a randomized control trial comparing three groups (n = 240), one in which mothers were encouraged to provide skin-to-skin care, one in which mothers were encouraged to provide an auditory-tactile-visual-vestibular (ATVV) intervention, and a control group, mothers in the skin-to-skin group experienced a quicker reduction in maternal worry [31]. Mothers in the ATVV group who provided infant massage experienced a significant decrease in maternal depression symptoms. Mothers in both the skin-to-skin and ATVV groups experienced less parenting stress compared to the control group.

In a crossover study of 28 parent-infant sets, associations among skin-to-skin holding, parental stress and anxiety, and oxytocin levels, oxytocin appears to have modulated stress and anxiety for both mothers and fathers [40]. Mothers and fathers were assigned skin-to-skin holding patterns in the NICU over a 2 day period. Parents' saliva was collected and anxiety was assessed using a validated scale before, during, and after skin-to-skin holding. Parents who participated in skin-to-skin holding experienced increased levels of oxytocin during the activity, and skin-to-skin holding was related to decreased stress and anxiety for both mothers and fathers. Interestingly, mothers and fathers also appeared to have synchronized levels of stress and anxiety in the NICU, which suggests that more research is needed on *all* caregivers' experiences in the NICU. Vittner et al.'s work supports these findings in their randomized cross-over study of 28 parent-infant sets, showing that parents who participate in skin-to-skin holding experience a rise in oxytocin levels and lower anxiety levels when measured across 3 days during a 60 minute holding session and again 45 minutes post-holding [26].

However, the relationship between skin-to-skin holding and maternal stress may need more contextual exploration. In a randomized control trial of 40 mother-infant dyads, mothers were given information about the NICU visitation policy and encouraged to skin-to-skin hold their infants. Mothers in the control group were also encouraged to blanket hold their infants at least 3 times a week for 50 minutes, while mothers in the intervention group were encouraged to skin-to-skin hold at least 3 times a week for 50 minutes. Maternal stress was measured using a validated scale once within 24 hours of NICU admission and then again within the 24 hours prior to NICU discharge. Results showed that hours of skin-to-skin holding were associated with increased maternal stress so that mothers who participated in more hours of skin-to-skin holding had higher stress levels [41]. When comparing the two groups, maternal stress was not significantly different between groups. Notably, this study was under-powered. A more recent randomized control trial examined 49 mothers of preterm infants in the NICU who had quit smoking during pregnancy. Participants were randomly assigned into either a control group or intervention group. Both groups were encouraged to remain smoke free and breastfeed, but the intervention group was supported in recognizing infant behavioral cues and skin-to-skin holding [35]. While results found participation in the control group was associated with smoking cessation and increased breastfeeding, there were no significant differences in maternal depression and stress scores between the two groups. These findings call for additional research in this area.

Another noteworthy finding is that maternal skin-to-skin holding has also been linked to substance use outcomes among mothers. In addition to the above finding that skin-to-skin holding may be associated with smoking cessation, mothers who identified as substance using in the mixed methods study described above in depth reported that participating in skin-to-skin holding motivated them to follow custody requirements and visit their infant more in the NICU [36].

### 3.5. Maternal-child bonding outcomes

Multiple studies examined how various maternal engagement activities may or may not relate to maternal-child bonding outcomes. Maternal visitation was found to be associated with mothers' perceptions of their infants' behavior and their infants' prognoses in a randomized control trial of 32 mother-infant dyads [27]. Mothers with higher visitation rates were less likely to have positive perceptions of their infants as measured by the “Your Baby” subscale on the Neonatal Perception Inventory. This finding may result from mothers having more realistic perceptions of behavior the more familiar they are with their infant. Mothers with higher visitation rates were more likely to have positive perceptions of their infant's prognoses, showing higher scores on measures of future intellectual and medical development.

Maternal skin-to-skin holding also showed positive maternal-child bonding outcomes. In a qualitative study, 10 mothers reported that skin-to-skin holding their preterm infant in the NICU facilitated their sense of familiarity with their infants' breathing patterns and preferred sleeping positions [39]. Skin-to-skin holding may also be linked to respiratory co-regulation between mothers and infants, as shown by a study of 11 parent-infant dyads. This study examined respiratory markers (e.g., skin temperature, heart rate) of parents and infants during a control period of the infant resting in an incubator and then during skin-to-skin holding. Results show that parents' heart rhythm influences infants' respiration pattern [42]. Studies of oxytocin and cortisol levels among parents and their preterm infants in the NICU point to their potential relationship to the skin-to-skin holding experience. For instance, a study hypothalamic-pituitary-adrenocortical (HPA) system activity among of 20 maternal-infant dyads found that skin-to-skin holding may facilitate the co-regulation of cortisol levels between mothers and infants [43]. A subsequent randomized control trial of mother-infant dyads (n = 79) who were encouraged to skin-to-skin or traditional blanket hold found that significant co-regulation of cortisol levels did not exist after 60 minutes of holding between the two groups. However, those groups compared to a control did show lower cortisol levels compared to the control group not encouraged to skin-to-skin hold or blanket hold [44]. Amounts of skin-to-skin holding have been found to positively relate to salivary oxytocin levels for parents and their preterm infants [26]. These findings are consistent with a more recent randomized crossover study that found that skin-to-skin care results in raised oxytocin levels for both parents and infants (n = 150), as well as decreased cortisol levels for both parents and infants. The same study demonstrated that parents who initiated skin-to-skin holding within the first two weeks had higher levels of parent engagement at NICU discharge, as measured by the PREEMI parent survey [26]. Additionally, parents with high oxytocin levels during skin-to-holding periods displayed more responsiveness and synchronicity with their infants in the NICU as measured by videotaped parent-infant interactions independently scored by two reviewers using the Dyadic Mutuality Code [26].

Interventions that support multiple maternal engagement activities also suggest an advantageous link between maternal engagement and maternal-infant bonding outcomes. The Family Nurture Intervention, designed to encourage skin-to-skin holding, blanket holding, exchange of scent clothes, eye contact between mother and infant, and mother touching the infant while talking to them, resulted in mothers who demonstrated more responsive caregiving behaviors compared to those who did not receive the intervention in a randomized control trial (n = 65) [45]. A similar study compared the ATVV intervention to skin-to-skin holding in a randomized control trial conducted in 4 NICUs. This study found that infants (n = 26) who received the multi-sensory intervention had more positive engagement and disengagement behaviors when interacting with their parents than those in the skin-to-skin holding group. The authors point out that interventions that promote infant engagement and disengagement behaviors are important because parents then have more opportunities to adjust their responses in ways that facilitate bonding and attachment [46]. In a separate study, parents who were trained to provide music and multimodal stimulation visited their infants more than parents who did not receive the training [28]. They also demonstrated more appropriate responses to their preterm infants in the NICU compared to parents not trained in the intervention, however these findings were not maintained at one-month post-discharge follow-up [28].

### 3.6. Breastfeeding outcomes

Breastfeeding may also be related to maternal engagement activities, in terms of both milk volume mothers are able to produce and duration of breastfeeding practices. Skin-to skin holding has been positively related to milk volume [47],[48]. One study used repeated-measures analysis of variance to compare breastmilk volume between a group of mothers who skin-to-skin held and a group that did not over a 2-week period. Mothers who skin-to-skin held had linear increase in volume over the 2-week period compared to no significant change in the control group. A study conducted 10 years later affirmed these findings, showing that among 124 participants, milk volume was positively correlated with skin-to-skin holding. This study also demonstrated that skin-to-skin holding time may increase mothers' motivation to express milk [48]. In a randomized cross over study of mothers (n = 28) who quit smoking during pregnancy, mothers who were supported in skin-to-skin holding and recognizing infant behavioral cues were more likely to be breastfeeding at 8 weeks postpartum compared to mothers who did not receive the skin-to-skin and behavioral cue support [35]. This finding is supported by a more recent secondary analysis of a multi-site, randomized trial of breastfeeding practices at NICU discharge and post-discharge (n = 231). Mothers who skin-to-skin held their preterm infant in the NICU were more likely to breastfeed at discharge and post-discharge follow-up compared to mothers randomly assigned to either an auditory-tactile-visual-vestibular intervention group or a group who received information about preterm infant care [49].

## 4. Discussion

This survey of research on the relationship of maternal engagement with maternal and infant health outcomes in U.S. NICUs highlights several promising avenues for further investigation, while also exposing a number of gaps in the current literature. Clearly, more research on maternal engagement is needed, especially because extant research suggests that some infant and maternal outcomes are significantly related to various aspects of maternal involvement, but questions remain about what types of engagement are most salient, how they should be measured, and which outcomes are the best predictors of long-term health and well-being. Notably, there is far more research on skin-to-skin holding than there is on other types of parental engagement in the NICU. While skin-to-skin holding seems associated with a number of desirable outcomes for both infants and mothers, it is as yet unclear whether skin-to-skin holding is more effective as an intervention than other types of engagement, including blanket holding and parental massage.

Nearly all observational cohort studies in the preterm population include cohorts of infants at various ranges of gestational ages but few make comparisons across gestational age categories to determine whether the degree of prematurity and subsequent medical complications impacts maternal engagement. Additional research assessing the degree of prematurity as the primary exposure and not just as a covariate for multivariate models may be needed to demonstrate the magnitude of its effect.

Research that examines the relationship between maternal involvement and infant health outcomes suggests that maternal engagement may have several positive effects on infant health. In particular, there is evidence to support an association of maternal engagement with infant breathing, temperature, and heart rate regulation, and with neurobehavioral outcomes such as crying behaviors. However, findings were mixed in explorations of associations between maternal involvement and NICU length of stay and a variety of post-discharge outcomes. Some of these variations in findings may have to do with how engagement was measured, including which activities were included in the construct and what methods were used for measuring levels of engagement. Some variation may have to do with differences in populations studied or study design.

Existing research also suggests that maternal engagement in the NICU may have important benefits for mothers themselves, in terms of both physiological and mental health outcomes. Importantly, research suggests that the benefits of skin-to-skin care are not limited just to mothers and infants, but also extends to fathers. However, mixed findings of other studies indicate that, at a minimum, more research is needed to understand the relationship between parental engagement of various forms and parental stress. Research should also examine the influence of engagement on other parental outcomes such as parental quality of life.

Perhaps some of the strongest research on maternal engagement relates to the effects of engagement on breastfeeding and smoking cessation. Breastfeeding and smoking cessation among mothers are strongly associated with positive infant health outcomes [50]–[55], and so the findings that maternal engagement through skin-to-skin holding in the NICU can promote these other beneficial maternal health behaviors is important. However, questions remain about which types and how much maternal engagement are necessary for these positive effects.

As this integrative review of existing literature indicates, research on the specific benefits of maternal engagement in the care of hospitalized preterm infants suggests that it may have promise as a strategy to improve infant health and developmental outcomes. However, further research is needed to define what behaviors and activities comprise “maternal engagement” in order to enhance family-centered care efforts. At the same time, more evidence is needed to understand what types of maternal engagement are most salient, and whether these types of involvement are also associated with positive outcomes for mothers.

One major area of study lacking from this literature thus far is an examination of what factors serve as facilitators and barriers to maternal involvement. It may be that factors external to the NICU environment, such as the need to balance work and other family obligations with NICU time, impact both how much time mothers can spend in the NICU, and how engaged they feel while there. The availability of financial resources that allows families to cover the cost of care of other children, delay return to work for parents with high risk infants, and pay for transportation to the NICU may be a significant barrier to engagement in the NICU [25],[56].

While these factors external to the NICU may be difficult to address in a short time frame, studies have demonstrated several aspects of NICU care that may increase parental engagement. For example, consistent primary nursing care, effective communication among providers and between providers and parents as well as greater parental education about their infants' medical conditions can lead to improvement parental engagement and satisfaction [56]–[58]. Thus, there exists “low hanging fruit” of potential interventions that can be adopted by hospitals to better engage parents in infant care.

Lastly, there are mixed findings related to quantity of time spent in the NICU, with some studies showing efficacy of intervals as short as 15 minutes of engagement. Thus, more testing should identify the most effective maternal involvement “dosage,” as well as the most effective forms of engagement during that engagement. Future research can help to clarify how mothers can best be involved in care that improves outcomes for infants and their mothers.

### 4.1. Limitations

This review offers important insights into the state of knowledge in this important area of inquiry. Because of the variance in the types of measures used in the studies examined here, a meta-analysis to evaluate overall strength of statistical relationships was not possible. As the research in this area matures, and measures and constructs become more standardized, meta-analytic techniques should be used to understand which types of maternal engagement are most salient for improved infant and maternal health.

## 5. Conclusion

A shift to family-centered care is underway in NICUs across the U.S., and with this shift comes increased attention toward facilitating quality engagement between hospitalized preterm infants and their parental caregivers. However, as this integrative review reveals, there is still much to learn about which types of engagement are more effective in boosting infant health outcomes, and what benefits these types of engagement might also have for mothers or other caregivers. Furthermore, there is wide variation in which outcomes are viewed as the most critical goals of interventions designed to enhance maternal engagement. It may be that improvements in pre-discharge clinical outcomes do not translate into long-term health benefits, or that the long-term benefits are best promoted through specific types of maternal engagement, particularly those that include education and training of mothers in skills needed to promote health at home.

## Conflict of interest

All authors declare no conflicts of interest in this paper.
